# Late relapse of non-seminomatous testicular cancer during treatment of multiple sclerosis with interferon β-1a: A case report

**DOI:** 10.3892/ol.2014.2519

**Published:** 2014-09-10

**Authors:** ISABEL BLANCAS, NURIA CÁRDENAS, MAYTE DELGADO, JOSE MIGUEL JURADO, MARTA LEGEREN, ANA VILLAESCUSA, FERNANDO GALVEZ, MARISOL YELAMOS

**Affiliations:** Oncology Unit, Hospital Clinico San Cecilio, Granada 18012, Spain

**Keywords:** inmunosupression, non-seminomatous germ cell tumor, late relapse, β-interferon, multiple sclerosis

## Abstract

Germ cell tumors (GCTs) comprise 95% of malignant tumors arising in the testes. The present study reports a patient diagnosed with non-seminomatous testicular cancer, stage IB, with a good risk prediction according to the International Germ Cell Cancer Collaborative Group classification. The patient received chemotherapy with bleomycin, etoposide and cisplatin, and achieved complete remission. Eleven years later, while receiving treatment with interferon β-1a for multiple sclerosis, the patient developed a relapse of the original cancer in the lungs and lymph nodes. The majority of GCTs relapse within the first two years of treatment, while 2–4% of patients can present with late relapses. There is no clear established association between multiple sclerosis and testicular cancer; we present the hypothesis that the inmunosupressor treatment that was administered for the multiple sclerosis promoted the cancer relapse.

## Introduction

Germ cell tumors (GCTs) are significant in malignant disease due to their increasing incidence, which has more than doubled in the past 40 years worldwide. GCTs constitute the most common type of solid tumor in males between the ages of 15 and 34 years. In total, 95% of malignant tumors arise in the testes, however, GCTs are relatively uncommon and comprise only 2% of all human malignancies (1).

The most common symtom is a painless solid testicular mass, but patients may present with persistent tenderness, swelling or another palpable abnormality.

A rapid diagnosis is important as a delay in diagnosis has been found to correlate with a higher stage at presentation (1). The primary treatment is an inguinal orchiectomy and the optimal care is based on histology, as well as the diagnosis of seminoma or non-seminoma, and stage. Sperm banking should be offered to patients prior to any therapeutic intervention that may compromise fertility. A careful evaluation of the patient must also be performed in order to determine if additional treatment is required, such as chemotherapy, radiotherapy or surgery.

The outcome is generally good, with a >90% cure rate of GCT patients, including a 70–80% cure rate for patients with advanced tumors who are treated with chemotherapy (1).

There is no clear established association between multiple sclerosis (MS) and its treatment and testicular cancer. For instance, Lebrun *et al* (2) reviewed >7,000 patients diagnosed with MS in order to assess whether there was an increase in the frequency of cancer incidence in these patients; however, noted a negative association was observed. There is, however, a well known relationship between MS and lymphoproliferative syndromes, such as Hodgkin’s lymphoma and infection by Epstein-Barr virus (3).

The majority of GCTs are known to relapse within the first two years of treatment, and 24% of patients can present late relapses with poor prognosis (4). In the present patient it is noteworthy that the relapse coincided with the administration of interferon β-1a for 10 months, for recently diagnosed MS.

The present study presents a patient with non-seminomatous testicular cancer who had a late relapse during treatment with interferon β-1a for MS. Patient provided written informed consent.

## Case report

### Presentation of disease

The present study reports the case of a 22-year-old male who presented to the to a Urology Unit of the Valley Hospital (Ridgewood, NJ, USA) with no significant previous clinical history before he noted a left testicular mass. Baseline significant findings were β-human chorionic gonadotropin (b-HCG) levels of 58.4 UI/l (normal range, <10 UI/l) and α-fetoprotein (AFP) levels of 73.7 ng/ml (normal range, <2 ng/ml). A computerized tomography (CT) scan of the chest, abdomen and pelvis was normal. Left inguinal orchiectomy was performed and subsequent analysis showed mixed germ cells, composed of embryonal carcinoma, mature teratoma and seminoma. Vascular invasion was present in the spermatic cord. The diagnosis and treatment classification was non-seminomatous germinal tumor (NSGT) E-IB, with a good risk prediction according to the International Germ Cell Cancer Collaborative Group classification (5). Six weeks after surgery, serological markers were within normal limits, and it was decided, in agreement with the patient, to conduct a strict follow-up procedure.

### Early relapse

At the two-month follow-up appointment, the CT scan showed an adenopathy of 2.2 cm in the left hilar renal zone. A retroperitoneal lymphadenectomy with removal of the remnant of the spermatic cord rendered one hilar renal lymph node and two paraaortic lymph nodes with metastasis of the original germinal tumor; the remainder of the sample was free of disease. The patient received two cycles of chemotherapy (QT) with bleomycin, etoposide and cisplatin (BEP). The patient’s progress was good and the patient continued yearly follow-up with no evidence of relapse.

### Diagnosis of MS

Eleven years later, at the age of 33 years, the patient underwent neurological consultation due to a sudden loss of visual acuity. Cranial magnetic resonance imaging and a multipotential study showed findings that were consistent with MS. The patient initially received high doses of steroids with limited clinical improvement. After suffering a relapse, the patient was treated with subcutaneous interferon β-1a at a dose of 44 mcg three days per week, and demonstrated favorable clinical evolution.

### Late relapse

Following 10 months of treatment with interferon β-1a, routine AFP levels showed an increase to 35.1 ng/ml (previous AFP levels were normal). b-HCG levels remained within the normal range (<0.1 UI/l), while the CT scan showed no tumor. A positron emission tomography (PET) scan, however, showed increased uptake in the left supraclavicular region and in both lungs. In view of these results and the stable neurological status, treatment with interferon β-1a was suspended.

A range of treatments were administered for this relapse; details of these treatments and the responses obtained are listed below. The patient underwent two cycles of BEP therapy, which were followed by disease progression. Subsequently, chemotherapy with methotrexate, vincristine, bleomycin, cisplatin, VP-16 and ifosfamide (BOMP-EPI) was administered, yielding a complete response after two cycles. The patient was then considered for intensive chemotherapy and autologous stem cell support. This option was declined by the patient and, thus, an additional BOMP-EPI cycle was given as consolidation. After three months, cervical adenopathy was detected by PET and confirmed by fine needle biopsy (necrosis and malignant cells compatible with testicular origin). Radiotherapy (44 Gy) was subsequently administered, but the patient refused any further treatment. Additional nodes and pulmonary metastasis were noted six months later. The patient then resumed treatment, which comprised gemcitabine and initially yielded a good response. However, novel pulmonary progression was noted on a follow-up CT scan. Following this, taxol, ifosfamide and carboplatin therapy was administered, with cisplatin substituted for carboplatin. A slow response and pulmonary stabilization became apparent after the third cycle, but was followed by worsening clinical and serological marker progression (AFP levels, 1,611 ng/ml). Rescue therapy with gemcitabine and oxaliplatin was then provided, but achieved no response, and subsequent treatment with etoposide also did not result in a response. After three months, the patient was admitted to hospital with an acute alteration in the level of consciousness that evolved into a coma; a large cerebral hemorrhage was observed and after 48 h, with no neurological improvement despite intensive treatment, the patient succumbed.

## Discussion

We conducted a search of the literature to determine whether an association between MS or its treatment with interferon β-1a and testicular cancer has been reported. However, no such previous study could be identified.

Several findings support the theory that MS is an immunologically mediated disorder involving one or more antigens located in the myelin of the central nervous system. Frohman *et al* (6) described how autoreactive T cells can cause inflammatory demyelination of the central nervous system. There appears to be a B-cell proliferation in the cerebrospinal fluid together with an increased number of mutations in B-cell receptors. This process is called somatic hypermutation, suggesting that a B-cell response to a specific antigen is occurring only in the central nervous system, as corresponding clones are absent from the pheripheral circulation. In addition, Levin *et al* (7) reported that T lymphocyte responses against myelin antigens are likely to be early contributors to the pathogenesis of MS. The authors also indicated that antibodies against myelin antigens exhibit a much more inconsistent association with the pathogenesis of MS, and that antibodies against non-myelin antigens, including neurofilaments, neurofascin, RNA binding proteins and potassium channels, may contribute to the pathogenesis of MS.

Consequently, immunomodulators and immunosuppressors are routinely used as the mainstay treatment for MS (6–8). This lead us to consider the possibility of an increase in tumor reactivation, associated with either the immunological alteration that causes MS or the iatrogenic suppression produced by the treatment, in the present case.

Interferon β-1a inhibits the synthesis and transport of matrix metalloproteinases and blocks antigen presentation (8). The CAMMS223 Trial Investigators *et al* (9) reported the appearance of colon cancer in association with interferon β-1a used as treatment for MS. Additionally, Burkitt lymphoma, breast cancer and cervical cancer *in situ* were observed to occur more frequently in association with alemtuzumab. In this study, the exclusion criteria were previous disease-modifying treatments, a history of clinically significant autoimmunity or the presence of serum antithyrotropin-receptor antibodies. There was no mention of previous cancer history.

Mullen *et al* (10) reported a case of melanoma in association with natalizumab treatment in a patient with MS. Natalizumab had been approved by the FDA for treatment of MS, but was later withdrawn from the market in February 2005, amid growing concerns of the risk of progressive multifocal leukoencephalopathy, to allow further assessment (2).

In the present case, there was concern regarding whether administering only two initial BEP cycles had been adequate. The disease had been classified initially as having a good prognosis (5). It has been seen, after long-term follow-up, that there are no statistically significant differences in survival between patients that received three or four cycles of BEP and had a germ-cell carcinoma with a favorable prognosis at the time of diagnosis (11). However, there is no data comparing the effect of two and three cycles, raising concern over whether the patient in the present case was undertreated.

GCTs represent one of the most rapidly proliferating tumors. The majority of patients relapse following surgical resection or chemotherapy-induced complete remission, whereby the relapse commonly occurs within two years of completion of therapy. Late relapse beyond two years is considered rare. Baniel *et al* (12) analyzed a large group of patients with testicular germ cell cancer in complete remission, who relapsed more than two years after the completion of treatment. Primary tumor cell type and elevated markers were not statistically associated with late relapse. The only factor found to be of prognostic value was the type of tumor cell at late relapse. Patients who relapse with malignant histology appear to have a 12-fold higher risk of mortality than those who recur with teratoma. Notably, a single factor, elevated AFP levels at first and second relapse, was associated with a higher risk of having a second or third relapse, as with the patient in the present case.

The subgroup of patients with late relapses is considered to have an unfavorable prediction and often present with ancestral cell types that are chemoresistant (12–16). A recent pooled analysis suggested higher late relapse rates for patients with non-seminoma (3.2%) than those with seminoma (1.4%). Bulky retroperitoneal lymphadenopathy and teratoma following chemotherapy may also portend a higher risk of relapse. Several characteristic features of late relapse are different from early relapse and initial disease presentations. These include a preponderance of yolk sac histology and abnormal elevation of AFP compared with b-HCG. However, in this study, there was no data regarding the type of tumor cell at relapse.

The patient in the present case was treated with the most common options that are contemplated in this situation. Interferon β-1a had been withdrawn at the moment of diagnosis and, thus, the patient was not treated as immunosuppressed. It is known that patients with immunosuppression (due to human immunodeficiency virus or following a transplant) are treated with the same protocols as those for immunocompetent individuals (17), with no significant differences in outcome.

The salvage treatments used for relapses include agents such as platinum, vinca, paclitaxel, ifosfamide, etoposide or gemcitabine (18–20). High-dose chemotherapy has not shown any benefit over conventional protocols. Several reviews show a tendency towards improved survival with dose-intensive treatment protocols, making it an option that should be taken into account (21–23). Multiple studies have demonstrated the importance of resecting residual masses following first-line or salvage chemotherapy for non-seminoma GCTs (12,24). However, in the present case, surgery was not an option due to the multiple lung metastasis. In conclusion, at any rate, cautious follow-up must be exercised when carrying out immunomodulatory treatment in a patient with a prior malignancy.

## References

[1] Motzer RJ, Agarwal N, Beard C (2009). NCCN clinical practice guidelines in oncology: testicular cancer. J Natl Compr Canc Netw.

[2] Lebrun C, Debouverie M, Vermersch P (2008). Cancer risk and impact of disease-modifying treatments in patients with multiple sclerosis. Mult Scler.

[3] Habek M, Brinar VV, Hajnsek S (2008). The association of multiple sclerosis and Hodgkin’s disease: the role of Epstein-Barr virus infection. Mult Scler.

[4] Ehrlich Y, Baniel J (2007). Late relapse of testis cancer. Urol Clin North Am.

[5] International Germ Cell Cancer Collaborative Group (IGCCCG) (1997). a prognostic factor-based staging system for metastatic germ cell cancers. J Clin Oncol.

[6] Frohman EM, Racke MK, Raine CS (2006). Multiple sclerosis - the plaque and its pahogenesis. N Engl J Med.

[7] Levin MC, Lee S, Gardner LA (2013). Autoantibodies to non-myelin antigens as cotributors to the pathogenesis of multiple sclerosis. J Clin Cell Immunol.

[8] Bellavista E, Santoro A, Galimberti D (2014). Current understanding on the role of standard and immunoproteasomes in inflammatory/immunological pathways of multiple sclerosis. Autoimmune Dis.

[9] Coles AJ, Compston DA, CAMMS223 Trial Investigators (2008). Alemtuzumab vs. Interferon beta-1a in early multiple sclerosis. N Engl J Med.

[10] Mullen JT, Vartanian TK, Atkins MB (2008). Melanoma complicating treatment with natalizumab for multiple sclerosis. N Engl J Med.

[11] Saxman S, Finch D, Gonin R (1998). Long-term follow-up of a phase III study of three versus four cycles of bleomycin, etoposide, and cisplatin in favorable-prognosis germ-cell tumores: the Indiana University Experience. J Clin Oncol.

[12] Baniel J, Foster R, Gonin R (1995). Late relapse of testicular cancer. J Clin Oncol.

[13] Oldenburg J, Martin JM, Fosså S (2006). Late relapses of germ cell malingnacies: incidence, management, and prognosis. J Clin Oncol.

[14] Sonpavde G, Huston TE, Roth BJ (2007). Management of recurrent testicular germ cell tumors. Oncologist.

[15] Feldman D, Bosl G, Sheinfeld J, Motzer RJ (2008). Medical treatment of advanced testicular cancer. JAMA.

[16] Ehrlich Y, Rosenbaum E, Baniel J (2013). Late relapse of testis cancer. Curr Urol Rep.

[17] Leibovitch I, Baniel J, Rowland RG (1996). Malignant testicular neoplasms in inmunosuppressed patients. J Urol.

[18] Farmakis D, Pectasides M, Pectasides D (2005). Recent advances in conventional-dose salvage chemotherapy in patients with cisplatin-resistant or refractory testicular germ cell tumors. Eur Urol.

[19] Germá-Lluch JR, García del Muro X, Tabernero JM (1999). BOMP/EPI intesive alternating chemotherapy for IGCCC poor-prognosis germ-cell tumors: the Spanish Germ-Cell Cancer Group experience (GG). Ann Oncol.

[20] De Giorgi U, Rosti G, Aieta M (2006). Phase II study of oxaliplatin and gemcitabine salvage chemotherapy in patients with cisplatin-refractory nonseminomatous germ cell tumor. Eur Urol.

[21] Schmoll HJ, Kollmannsberger C, Metzner B (2003). Long-term results of first-line sequential high-dose etoposide, ifosfamide, and cisplatin chemotherapy plus autologous stem cell support for patients with advanced metastatic germ cell cancer: an extended phase I/II study of the German Testicular Cancer Study Group. J Clin Oncol.

[22] Ayash LJ, Clarke M, Silver SM (2001). Double dose-intensive chemotherapy with autologus stem cell support for relapsed and refractory testicular cancer: the University of Michigan experience and literature review. Bone Marrow Transplant.

[23] Miki T, Mizutani Y, Akaza H (2007). Long-term results of first-line sequential high-dose carboplatin, etoposide and ifosfamide chemotherapy with peripheral blood stem cell support for patients with advanced testicular germ cell tumor. Int J Urol.

[24] Geldart TR, Gale J, Mckendrick J (2006). Late relapse of metastatic testicular nonseminomatous germ cell cancer: surgery is needed for cure. BJU Int.

